# Association Between Postoperative Long-Term Heart Rate Variability and Postoperative Delirium in Elderly Patients Undergoing Orthopedic Surgery: A Prospective Cohort Study

**DOI:** 10.3389/fnagi.2021.646253

**Published:** 2021-05-31

**Authors:** Jiaduo Sun, Qingguo Zhang, Baojia Lin, Mengjiao He, Yimin Pang, Qibo Liang, Zhibin Huang, Ping Xu, Dongdong Que, Shiyuan Xu

**Affiliations:** ^1^Department of Anesthesiology, Zhujiang Hospital, Southern Medical University, Guangzhou, China; ^2^Department of Cardiology, Zhujiang Hospital, Southern Medical University, Guangzhou, China

**Keywords:** heart rate variability, postoperative delirium, autonomic nervous system, elderly patients, orthopedic surgery

## Abstract

**Background:**

Postoperative delirium (POD) is a common complication after orthopedic surgery in elderly patients. The elderly may experience drastic changes in autonomic nervous system (ANS) activity and circadian rhythm disorders after surgery. Therefore, we intend to explore the relationship between postoperative long-term heart rate (HR) variability (HRV), as a measure of ANS activity and circadian rhythm, and occurrence of POD in elderly patients.

**Methods:**

The study population of this cohort was elderly patients over 60 years of age who scheduled for orthopedic surgery under spinal anesthesia. Patients were screened for inclusion and exclusion criteria before surgery. Then, participants were invited to wear a Holter monitor on the first postoperative day to collect 24-h electrocardiographic (ECG) data. Parameters in the time domain [the standard deviation of the normal-to-normal (NN) intervals (SDNN), mean of the standard deviations of all the NN intervals for each 5-min segment of a 24-h HRV recording (SDNNI), and the root mean square of successive differences of the NN intervals (RMSSD)] and frequency domain [heart rate (HR), high frequency (HF), low frequency (LF), very low frequency (VLF), ultra low frequency (ULF), and total power (TP)] were calculated. Assessment of delirium was performed daily up to the seventh postoperative day using the Chinese version of the 3-Min Diagnostic Interview for CAM-defined Delirium (3D-CAM). The relationship between HRV and POD, as well as the association between HRV and duration of POD, was assessed.

**Results:**

Of the 294 cases that finally completed the follow-up, 60 cases developed POD. Among the HRV parameters, SDNNI, VLF, and ULF were related to the occurrence of POD. After adjustment for potential confounders, the correlation between HRV indices and POD disappeared. Through stratified analysis, two significant negative correlations emerged: ULF in young-old participants and SDNNI, VLF, and ULF in male patients.

**Conclusion:**

The lower HRV parameters may be related to the occurrence of POD, and this correlation is more significant in young-old and male patients. ANS disorders and rhythm abnormalities reflected by HRV changes may represent a possible mechanism that promotes POD.

## Introduction

Delirium is a neurocognitive disorder characterized by a disturbance in attention, level of consciousness, and cognition, in which symptoms are acute in onset and may fluctuate in severity throughout the day (Battle, 2013). Postoperative delirium (POD) is common after elective orthopedic surgery, with an estimated incidence of 17.3% (Scott et al., 2015), and is associated with significant adverse outcomes, including dementia and institutionalization (Inouye et al., 2014). Orthopedic surgery is a common surgery in patients over 60 years old. As the latest official statistics shows that there are more than 250 million people over the age of 60 in China (National Bureau of Statistics of China, 2020), the demand for hip fracture surgery and total joint arthroplasty is expected to rise dramatically (Kurtz et al., 2007; Zhang et al., 2020). Although age and prior cognitive impairment are risk factors for POD (Dasgupta and Dumbrell, 2006; van Meenen et al., 2014), the underlying pathophysiology of delirium is still poorly understood (Maldonado, 2013). As is proved acting in many mental disorders (Kim et al., 2018; Mulcahy et al., 2019), alterations in autonomic nervous system (ANS) activity have been suggested to be related to delirium (Maclullich et al., 2008). Besides, circadian rhythm that is regulated by the complex interaction between the central nervous system (CNS) and the ANS has also been demonstrated to be related to POD (Maldonado, 2018; Riganello et al., 2019a,b).

Specifically, both ANS activity and circadian rhythm can indirectly be assessed by measuring heart rate variability (HRV) non-invasively (Shaffer et al., 2014; Shaffer and Ginsberg, 2017; Vitale et al., 2019). HRV is the fluctuation in the time intervals between adjacent heartbeats, which indexes neurocardiac function and is generated by heart–brain interactions and dynamic non-linear ANS processes (Singh et al., 2018b). Because of the advantages of economy and ease of recording, HRV measurement is particularly applicable in studies on large subject samples.

Heart rate variability measurement mainly includes two methods: short-term (5 min) and long-term (24 h). The first method is obtained by spectral analysis of the electrocardiographic (ECG) data obtained within a 5-min period. It is recommended to record in laboratory conditions before and after tilt, drugs, controlled ventilation, or other maneuvers selected to challenge the ANS. In the second method, HRV is determined from long-term ECG recordings, traditionally 24 h. In particular, a longer recording (24 h) better represents the daily activity, the response to environmental stimuli, and the central nervous activity including circadian rhythms (No authors listed, 1996; Shaffer and Ginsberg, 2017; Singh et al., 2018a,b).

Although studies have shown that HRV is associated with cognitive function in older patients (Kim et al., 2018; Dalise et al., 2020), there are few studies concerning the correlation between long-term HRV parameters and POD. As is proved in previous researches, HRV decreases to varying degrees in elderly patients after surgery, indicating that ANS activity and circadian rhythm were impaired after surgery (Marsch et al., 1994; Amar et al., 1998; Kärkelä et al., 2002). Previous studies tend to explore the association between preoperative short-term HRV parameters and POD. Predictably, due to its high temporal resolution, the long-term HRV measured postoperatively may reflect changes in ANS activity and circadian rhythm after surgery more accurately.

Thus, this prospective cohort study intends to explore the relationship between postoperative long-term (24 h) HRV and the occurrence of POD.

## Materials and Methods

### Subject Recruitment

This prospective cohort study was conducted in the Zhujiang Hospital of Southern Medical University from June 1, 2019, to October 30, 2020. The study protocol was approved by the Institutional Committee for Medical Ethics (approval number 2020-KY-065-02, Medical Ethics Committee of Zhujiang Hospital of Southern Medical University, China). Patients were asked to give written informed consent to participate. We enrolled patients aged 60 or older who scheduled for orthopedic surgery [including total hip arthroplasty (THA), total knee arthroplasty (TKA), revision TKA (RTKA), revision THA (RTHA), hip fracture repair, and femoral shaft fracture (FSF) surgery] under spinal anesthesia. Exclusion criteria were as follows: (I) American Society of Anesthesiologists (ASA) grade ≥ IV; (II) clinical evidence of acute coronary artery disease in the last 3 months, significant valve disease; (III) arrhythmia (atrial fibrillation or flutter, ectopic beat > normal sinus 5% of pulsation); (IV) β-blocker user; (V) history of endocrine disease (thyroid or adrenal gland disease); (VI) Parkinson disease (PD), all-cause dementia (including PD-related dementia, AD-related dementia, and Lewy’s body dementia), CNS tumor, and stroke with hemiplegia; (VII) preoperative mini-mental state examination (MMSE) score <17 or preoperative delirium; (VIII) severe hearing impairment and inability to communicate; (IX) long-term usage of sedatives or other mental illness drugs, and drug or alcohol abuse; and (X) transferred to an intensive care unit (ICU) postoperatively.

### Measurement of Postoperative Heart Rate and Heart Rate Variability

With the end of the operation, participants were invited to wear a 12-lead Holter monitor (Biomedical Instruments Limited Company, Shenzhen, China) and then transferred back to quiet inpatient wards for 24 h. In order to prevent movement from interfering with HRV, during the recording process, participants were asked to relax as much as possible and lie in bed for a long time.

Holter monitor recordings were transmitted to the Dynamic ECG analysis system (Biomedical Instruments Limited Company, Shenzhen, China) and interpreted using the same software. HRV parameters were automatically documented as numerical data by the Holter software. Artifacts and arrhythmia were manually identified and removed by an experienced cardiologist blinded to the clinical data of the cases. Only the analyzable ECG data recorded for more than 18 h could be considered qualified.

Parameters in time domain and frequency domain were applied to HRV assessment. In the time domain, the following indices were calculated: SDNN (in milliseconds), SDNNI (in milliseconds), and RMSSD (in milliseconds). In the frequency domain, the power spectra of the following frequency bands were calculated: HR, HF (0.15–0.40 Hz, in normal units), LF (0.04–0.15 Hz, in normal units), VLF (0.0033–0.04 Hz, in milliseconds), ULF (≤0.003 Hz, in milliseconds), and TP (in milliseconds).

### Assessment of Delirium and Cognitive Scanner

We interviewed all patients the day before the operation. Patients were screened for dementia through an MMSE test and were recruited with a score of more than 16. The assessment of delirium was conducted using the Chinese version of the 3-Min Diagnostic Interview for CAM-defined Delirium (3D-CAM) by investigators who were trained and supervised by a delirium expert. The evaluation included face-to-face interviews with patients and their accompanying family members or nursing assistants and review of the medical records and nurse’s notes since patients’ admission.

The 3D-CAM has four diagnostic features: (1) an acute change and fluctuating course, (2) inattention, (3) disorganized thinking, and (4) altered level of consciousness. Delirium is suggested if both features 1 and 2 are present at the same time that either feature 3 or 4 is identified. In addition, the 3D-CAM was chosen to detect POD due to its high sensitivity in comparison with the gold standard, the *Diagnostic and Statistical Manual of Mental Disorders*, 5th edition (DSM5), criteria (Marcantonio et al., 2014). Moreover, the Chinese version has also been proven to have good reliability and validity with use in the Chinese elderly population (Ji et al., 2020).

For delirium patients, the Chinese version of the Delirium Rating Scale-98-R (DRS-98-R) was additionally used to assess the subtype of delirium. According to the scale, delirium can be classified into three motoric subtypes. The hyperactive subtype was defined as a score of 1 to 3 on Item 7 of the DRS-98-R (motor agitation) and a score of 0 on Item 8 of the DRS-98-R (motor retardation). The hypoactive subtype was defined as a score of 0 on Item 7 of the DRS-98-R and a score of 1 to 3 on Item 8 of the DRS-98-R. The mixed subtype was defined as a score of 1 to 3 on Items 7 and 8 in the DRS-98-R or a score of 0 on both items (Huang et al., 2009).

Investigators performed delirium assessments 1 day before the scheduled surgery and daily up to the seventh postoperative day from 7 am to 9 am. Patients with POD were be followed up until discharged. Both the subtype and duration of delirium were required to be recorded.

### Covariates

Studies had shown that age, Charlson Comorbidity Index (CCI), and perioperative usage of benzodiazepine and opiates were associated with delirium after orthopedic surgery in elderly patients (Scott et al., 2015; Weinstein et al., 2018). In addition, body mass index (BMI), sex, and physical status were correlated with HRV (Shaffer and Ginsberg, 2017). Therefore, the following variables were collected as potential confounders: age, sex, BMI, CCI, surgery, dosage of midazolam, and equivalent morphine usage.

### Demographic Characteristics, Lifestyle, and Clinical Assessment

Demographic characteristics (weight, height, BMI, sex, and educational level), lifestyle habits (mainly drinking and tobacco smoking), and clinical features (test results, diagnosis, past medical history, type of surgery, surgery duration, and concomitant medications) were collected through a purpose-designed questionnaire, administered by an interviewer, and through available medical records.

### Statistical Analysis

A minimum of 10 events per variable are necessary to adequately produce estimates of effect with regression models (Concato et al., 1995). Based on the reported incidence of POD after orthopedic surgery of approximately 17.3% (Scott et al., 2015), a sample size of 289 individuals will allow five variables to be assessed in the regression model.

Descriptive statistics were expressed as the mean ± SD, median (interquartile range), or frequencies with percentages to compare characteristics of participants with and without POD including demographics, behavioral habits, and outcomes. HRV parameters were compared between the groups stratified by the presence of POD using Mann–Whitney *U* tests. Analyses further involved Spearman’s rank correlation coefficients of the entire cohort to evaluate a potential relationship between HRV parameters and POD. Then, Spearman’s rank correlation coefficients were used to estimate the associations between the HRV parameters and the duration of POD. Furthermore, HRV parameters that differed between the groups at the 20% level of significance (i.e., *P* < 0.20) were entered into multivariable logistic regression models for the outcome of POD. Covariates were chosen based on significant associations with the occurrence of POD and previous literature. All analyses were conducted using the Statistical Package for the Social Sciences (SPSS) version 25.

## Results

### Study Population

**FIGURE 1:**
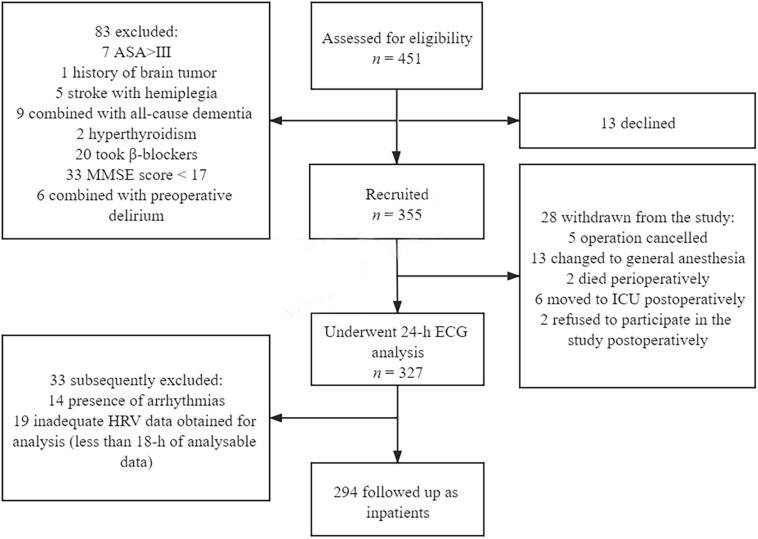
Flowchart of the study.

According to the exclusion criteria, 355 of the 451 patients were recruited between June 1, 2019, and October 30, 2020 (Figure 1). In the end, 294 patients completed follow-up. Sixty participants (20.4%) developed POD with a median duration of 1 day.

Characteristics of the cohort stratified by the presence of POD are shown in Table 1. Compared with patients not experiencing POD, delirium patients tended to be older and had a higher ASA PS, higher CCI, higher dosage of opiates, and higher percentage of hip surgery (including THA, RTHA, and hip fracture repair). They also had a lower preoperative hemoglobin (Hb) concentration and lower dosage of midazolam. In contrast, there were no differences in sex, education level, duration of surgery, drinking, and smoking between groups.

**TABLE 1:** Descriptive characteristics of the cohort, stratified by POD.

	No POD, *n* = 234 (79.6%)	POD, *n* = 60 (20.4%)	*P*
Age (years)	69.49 ± 7.43	73.9 ± 8.11	<0.001
Sex			0.249
female	177 (75.64%)	41 (68.33%)	
male	57 (24.36%)	19 (31.67%)	
Education level			0.973
<high school	175 (74.79%)	45 (75.00%)	
≥high school	59 (25.21%)	15 (25.00%)	
Smoking			0.541
yes	43 (18.38%)	9 (15.00%)	
no	191 (81.62%)	51 (85.00%)	
Drinking			0.077
yes	23 (9.83%)	2 (3.33%)	
no	211 (90.17%)	58 (96.67%)	
BMI, kg/m^2^	24.79 ± 4.46	23.97 ± 3.87	0.192
Hb (g/L)	124.62 ± 16.22	115.62 ± 18.55	<0.001
ASA PS			<0.001
1 or 2	200 (85.47%)	36 (60.00%)	
3	34 (14.53%)	24 (40.00%)	
CCI	0.64 ± 1.14	1.20 ± 1.33	0.004
Type of surgery			0.010
THA or RTHA	61 (26.07%)	20 (33.33%)	
TKA or RTKA	136 (58.12%)	22 (36.67%)	
Hip fracture repair	24 (10.26%)	14 (23.33%)	
FSF surgery	13 (5.55%)	4 (6.67%)	
Duration of surgery (min)	129.99 ± 48.37	133.50 ± 49.88	0.619
Midazolam (mg)	2.01 ± 0.76	1.57 ± 0.71	<0.001
Equivalent morphine (mg)	42.1 ± 48.63	72.61 ± 100.85	0.026
Duration of delirium (day)	–	1.00 (1.00, 2.00)	–

*Data are reported as mean ± SD, n (percentage), or median (interquartile range). BMI, body mass index; Hb, hemoglobin; ASA PS, physical status according to the American Society of Anesthesiologists; CCI, Charlson Comorbidity Index; THA, total hip arthroplasty; RTHA, revision total hip arthroplasty; TKA, total knee arthroplasty; RTKA, revision total knee arthroplasty; FSF surgery, femoral shaft fracture surgery; POD, postoperative delirium.*

In patients with POD, 18 were assessed as hyperactive subtype of delirium, 17 as hypoactive subtype, and 25 as mixed subtype. Except for the duration of surgery, there was no difference in the characteristics among three subtypes. Compared with the other two subtypes, the operation time was shorter in mixed subtype (Supplementary Table 1).

### Postoperative Delirium and Heart Rate Variability

Heart rate variability parameters of the cohort stratified by the presence of POD were expressed as median and reported in Table 2. Aside from RMSSD and HR, other HRV indices were higher in patients who experienced POD compared with those without delirium. These differences were significant for SDNNI (*P* = 0.027) and ULF (*P* = 0.006).

**TABLE 2:** HRV parameters of the cohort, stratified by POD.

	No POD, *n* = 234 (79.6%)	POD, *n* = 60 (20.4%)	*P*
Time for analysis (min)	1,428.00 (1,299.00, 1,440.00)	1,436.00 (1,351.00, 1,440.00)	0.295
HR mean (bpm)	73.54 ± 10.79	75.60 ± 12.88	0.258
SDNN (ms)	79.50 (62.00, 100.00)	72.50 (57.00, 104.50)	0.392
SDNNI (ms)	33.00 (24.00, 41.00)	29.00 (19.50, 37.00)	0.027
RMSSD (ms)	19.00 (14.00, 28.00)	20.00 (12.50, 26.50)	0.593
HF (nu)	36.03 (27.74, 47.04)	35.53 (28.95, 47.99)	0.929
LF (nu)	55.36 (47.58, 62.34)	53.94 (45.24, 60.11)	0.312
VLF (ms)	487.80 (272.50, 701.50)	356.05 (163.95, 505.10)	0.136
ULF (ms)	21.75 (12.50, 31.20)	15.15 (6.60, 23.25)	0.006
TP (ms)	795.90 (467.70, 1,215.10)	637.15 (265.80, 985.60)	0.941

*Data are reported as mean ± SD or median (interquartile range). HR, heart rate; bpm, beats per minutes; SDNN, standard deviation of all normal-to-normal intervals; SDNNI, mean of the standard deviations of all the normal-to-normal intervals for each 5-min segment of a 24-h HRV recording; RMSSD, square root of the mean of the sum of the squares of differences between adjacent normal-to-normal intervals; HF, high frequency; LF, low frequency; VLF, very low frequency; ULF, ultra-low frequency; TP, total power; POD, postoperative delirium.*

A comparison of HRV among three subtypes of delirium patients is illustrated in Supplementary Table 2. No differences were observed in the HRV indices among groups.

Table 3 shows the correlation analysis between HRV and POD. The occurrence of POD was significantly negatively correlated with SDNNI, VLF, ULF, and TP but not with SDNN, HF, or LF, which were traditionally of concern. As stratified into young-old (60–79 years old) and old-old (≥80 years old) participants by age, POD was still significantly negatively correlated with SDNNI, VLF, and ULF in young-old adults rather than in the old-old population (Supplementary Table 3).

**TABLE 3:** Spearman’s correlation coefficients between HRV parameters and POD.

	POD	
Variables	ρ	*P*
SDNN (ms)	–0.057	0.331
SDNNI (ms)	–0.142	0.015
RMSSD (ms)	–0.046	0.433
HF (nu)	0.030	0.603
LF (nu)	–0.053	0.367
VLF (ms)	–0.186	0.001
ULF (ms)	–0.185	0.001
TP (ms)	–0.128	0.028

*SDNN, standard deviation of all normal-to-normal intervals; SDNNI, mean of the standard deviations of all the normal-to-normal intervals for each 5-min segment of a 24-h HRV recording; RMSSD, square root of the mean of the sum of the squares of differences between adjacent normal-to-normal intervals; HF, high frequency; LF, low frequency; ULF, ultra-low frequency; TP, total power; HRV, heart rate variability; POD, postoperative delirium.*

Observed to be associated with cognitive function (Dalise et al., 2020), HF and LF were selected for logistic regression analyses. In addition, as SDNNI, VLF, and ULF were associated with POD at a significance level of less than 0.2, they were also included in the planned binary logistic regression analyses. After potential confounders were controlled for (i.e., sex, age, BMI, educational level, CCI, surgery, dosage of midazolam, and equivalent morphine), there was no significant association between HRV indices and POD (Table 4). Noticeably, after stratification by age, there was a significant association between ULF and POD in young-old adults (Table 5). Stratified by sex subsequently, SDNNI, VLF, and ULF were significantly associated with POD in male patients (Table 6).

**TABLE 4:** Adjusted regression coefficients (95% confidence interval) between HRV parameters and POD.

Variables	aOR	95% CI
SDNNI (ms)	0.976	(0.951, 1.003)
HF (nu)	0.987	(0.963, 1.011)
LF (nu)	1.003	(0.978, 1.029)
VLF (ms)	0.999	(0.998, 1.000)
ULF (ms)	0.976	(0.951, 1.002)

*Adjusted variables: sex, age, BMI, education, CCI, surgery, dosage of midazolam, and equivalent morphine. aOR, adjusted odds ratio; SDNNI, mean of the standard deviations of all the normal-to-normal intervals for each 5-min segment of a 24-h HRV recording; HF, high frequency; LF, low frequency; VLF, very low frequency; ULF, ultra-low frequency; HRV, heart rate variability; POD, postoperative delirium; BMI, body mass index; CCI, Charlson Comorbidity Index.*

**TABLE 5:** Adjusted regression coefficients (95% confidence interval) between HRV parameters and POD, stratified by age.

	Young-old, *n* = 251 (85.37%)		Old-old, *n* = 43 (14.63%)	
Variables	aOR	95% CI	aOR	95% CI
SDNNI (ms)	0.972	(0.943, 1.002)	0.980	(0.914, 1.051)
HF (nu)	0.993	(0.964, 1.023)	0.968	(0.924, 1.014)
LF (nu)	1.005	(0.974, 1.038)	1.003	(0.957, 1.051)
VLF (ms)	0.999	(0.998, 1.001)	0.998	(0.994, 1.002)
ULF (ms)	0.970	(0.942, 0.999)	0.981	(0.891, 1.079)

*Adjusted variables: sex, BMI, education, CCI, surgery, dosage of midazolam, and equivalent morphine. aOR, adjusted odds ratio; SDNNI, mean of the standard deviations of all the NN intervals for each 5-min segment of a 24-h HRV recording; HF, high frequency; LF, low frequency; VLF, very low frequency; ULF, ultra-low frequency; HRV, heart rate variability; POD, postoperative delirium; BMI, body mass index; CCI, Charlson Comorbidity Index.*

**TABLE 6:** Adjusted regression coefficients (95% confidence interval) between HRV parameters and POD, stratified by sex.

	Female, *n* = 218 (74.15%)		Male, *n* = 76 (25.85%)	
Variables	aOR	95% CI	aOR	95% CI
SDNNI (ms)	0.991	(0.962, 1.021)	0.927	(0.870, 0.987)
HF (nu)	0.981	(0.952, 1.010)	0.998	(0.954, 1.044)
LF (nu)	1.009	(0.979, 1.041)	0.993	(0.944, 1.044)
VLF (ms)	1.000	(0.999, 1.001)	0.997	(0.994, 0.999)
ULF (ms)	0.991	(0.963, 1.021)	0.929	(0.871, 0.991)

*Adjusted variables: age, BMI, education, CCI, surgery, dosage of midazolam and equivalent morphine. aOR, adjusted odds ratio; SDNNI, mean of the standard deviations of all the NN intervals for each 5-min segment of a 24-h HRV recording; HF, high frequency; LF, low frequency; VLF, very low frequency; ULF, ultra-low frequency; HRV, heart rate variability; POD, postoperative delirium; BMI, body mass index; CCI, Charlson Comorbidity Index.*

The median duration of POD was 1 day. There were significant negative correlations of SDNNI, VLF, ULF, and TP but not RMSSD, HF, or LF with the duration of POD (Table 7).

**TABLE 7:** Spearman’s correlation coefficients between HRV parameters and the duration of POD.

	POD duration	
Variables	ρ	*P*
SDNN (ms)	–0.064	0.271
SDNNI (ms)	–0.156	0.007
RMSSD (ms)	–0.057	0.331
HF (nu)	0.039	0.502
LF (nu)	–0.059	0.310
VLF (ms)	–0.205	<0.005
ULF (ms)	–0.204	<0.005
TP (ms)	–0.143	0.014

*SDNN, standard deviation of all normal-to-normal intervals; SDNNI, mean of the standard deviations of all the NN intervals for each 5-min segment of a 24-h HRV recording; RMSSD, square root of the mean of the sum of the squares of differences between adjacent normal-to-normal intervals; HF, high frequency; LF, low frequency; VLF, very low frequency; ULF, ultra-low frequency; TP, total power; HRV, heart rate variability; POD, postoperative delirium.*

## Discussion

To our knowledge, this study is the first prospective cohort study to explore the relationship between postoperative long-term HRV parameters and the occurrence of POD. According to results, we observed a connection between HRV indices and POD, which may be more pronounced in young-old patients.

In recent years, neuroimaging studies have shown a functional interaction between the ANS and the CNS, which are involved in the neural network of higher brain functions (including attention and consciousness processes) (Thayer et al., 2009; Riganello et al., 2019a,b). As generated by heart–brain interactions and dynamic non-linear ANS processes, HRV is used to represent ANS activity and circadian rhythm (Shaffer et al., 2014; Shaffer and Ginsberg, 2017; Vitale et al., 2019). Due to homeostatic requirements with different timing and latencies, CNS and ANS setups change over time spontaneously. As proven by previous studies, the elderly experienced drastic changes in ANS activity and circadian rhythm after surgery (Marsch et al., 1994; Amar et al., 1998; Kärkelä et al., 2002). Because of high time resolution during the day of the HRV measures, the purpose of our research is to explore the relationship between postoperative HRV and POD.

In this study, we observed that VLF, ULF, TP, and SDNNI were significantly negatively correlated with the occurrence of POD.

The VLF and ULF bands require a long-term recording, which may be best monitored over 24 h (Shaffer et al., 2014). Their band lie in the 95% of the total power of the HRV power in a 24-h recording (Fisher et al., 2014). VLF power is strongly associated with all-cause mortality (Tsuji et al., 1994; Hadase et al., 2004; Schmidt et al., 2005), post-traumatic stress disorder (PTSD) (Ulmer et al., 2018), and emotional stress (Fisher et al., 2014). Parasympathetic nervous system activity was found to contribute VLF power mainly (Berntson et al., 1997; Taylor et al., 1998).

Theoretically, ANS can participate in the regulation of inflammatory reflex through cholinergic anti-inflammatory pathway (Huston and Tracey, 2011; Williams et al., 2019). Low VLF power has been found to be associated with higher levels of inflammation in several studies (Carney et al., 2007; Lampert et al., 2008). Unbalanced inflammatory response and dysfunctional interaction between the cholinergic and immune systems can contribute to POD (Cerejeira et al., 2012; Riedel et al., 2014; Subramaniyan and Terrando, 2019). In our result, the lower VLF power may indicate more severe inflammatory response, and cholinergic dysfunction occurred in patients with POD. Also, over a 24-h period, VLF power is correlated with SDNNI, which reflects the global autonomic modulation of the heart (Almeida-Santos et al., 2016).

Except other factors (body temperature regulation and metabolism), the circadian oscillation in HR is the primary source of the ULF power (Laborde et al., 2017). Circadian rhythm disruption was deemed to contribute to the development of POD by itself or by interacting with pain and inflammation (Chouchou et al., 2014; Scott, 2015; Fadayomi et al., 2018). Therefore, the lower ULF indices may manifest circadian rhythm disruption of delirium patients.

Furthermore, movement is likely to impair the power of ULF and VLF (Hunt and Saengsuwan, 2018). According to the subtype of delirium (Liptzin and Levkoff, 1992; Meagher and Trzepacz, 2000), patients with hyperactive (characterized by agitation, aggression, hallucinations, and disorientation) or mixed (fluctuation between hypoactive and hyperactive subtypes) subtype may show increased movement. As our result showed, the lower ULF power and VLF power in patients with POD were consistent with clinical manifestation of delirium.

Crucially, delirium is considered a marker of the vulnerable brain with diminished reserve capacity predisposed in large part by advanced biological – not chronologic – age (Inouye et al., 2014). Previous researches have shown that long-term HRV indexes can be used as a biomarker of the aging process (Corino et al., 2006; Perseguini et al., 2015; Tan et al., 2019). Based on this, the connection between HRV indices and POD may also reflect the effect of aging on POD to a certain extent.

By means of regression analysis that included several relevant confounders, we did not find postoperative VLF, ULF, and SDNNI indices to be independently associated with the occurrence of POD. Noticeably, after stratification by age, there was a significant association between ULF and POD in young-old adults. Subsequently, after stratification by sex, results showed SDNNI, VLF, and ULF were significantly associated with POD in men instead of women. This may be due to a few different reasons.

The aging process is related to a reduction in the interaction ability of biological systems including the ANS (Lakatta and Levy, 2003; Niccoli and Partridge, 2012; Chadda et al., 2018). Multiple studies have shown that HRV decreases with age (Corino et al., 2006; Almeida-Santos et al., 2016; Botsva et al., 2017). However, it should not be ignored that some of the HRV indices show a non-linear decreasing pattern as age increased. Results of two cohort studies in Brazil showed that parasympathetic associated time domain variables showed a U-shaped distribution and reversal increase between 60 and 70 years old, which were consistent with trend of the frequency domain parameters (HF) changing with age in our cohort (Almeida-Santos et al., 2016; Geovanini et al., 2020; Supplementary Table 4). Theoretically, the VLF band, which contributed mainly by parasympathetic nervous system activity, may display a reversal process in old-old patients (Berntson et al., 1997; Taylor et al., 1998). Furthermore, the decrease of HRV with age is accompanied by changes in functional connectivity along the cortical midline (Kumral et al., 2019). It is foreseeable that there would be a complex-pattern HRV changing in old-old patients.

Therefore, we speculate that the complex non-linear interaction between aging and HRV may be the cause of the disappearance of the correlation between HRV indices and POD after adjusting for confounding factors including age. Instead of the old-old patients, the correlation between HRV indices and POD may be more significant in young-old patients.

As for the difference in results between different sexes, a previous cohort study of hip fractures displayed that men were more likely to experience POD than women (Oh et al., 2016). Male patients were observed to have higher scores on motor agitation and affective lability, whereas females have a higher frequency of hypoactive delirium (Trzepacz et al., 2018). In our result, although there is no statistical difference in gender among the three subtypes of delirium, the proportion of hyperactive subtype in delirium male patients was higher than that in females (42.1 vs. 24.3%). The characteristics of hyperactive delirium may exert an influence on HRV power. Alternatively, we had more women in this study population than men, and the difference may have been due to chance or unmeasured confounders. Therefore, it is worthwhile to further investigate the differences on age and gender.

Finally, we did not find any difference in HRV parameters among three subtypes of delirium. Clinically, delirium symptoms are acute in onset and fluctuate in severity throughout the day. The three subtypes of delirium can be transformed into each other during hospitalization. For example, mixed subtype cloud manifests hyperactive or hypoactive symptoms. Patients who experienced both hyperactive and hypoactive symptoms were assessed as mixed subtype. Therefore, the difficulty to define the subtype of delirium clearly may be the main reason.

### Comparison With Existing Literature

A survey in an ICU observed that the delirium patients had a lower LF/HF ratio and a greater reduction in SDNN than the control group during head-up tilt to 15° (Neerland et al., 2019). In our results, the frequency and time domain parameters of patients with POD were mostly lower, which was consistent with the result of a research about HRV at mild cognitive impairment stage of Lewy body disease (Kim et al., 2018).

Conversely, a research in patients with hip fracture showed that preoperative SDNN, TP, and HF values were significantly higher in patients with delirium and that LF and LF/HF were lower compared with those in patients without delirium (Ernst et al., 2020). One reason for this discrepancy might be as follows: this investigation studied HRV by analyzing short-term measurements based on preoperative 5-min recordings, which could not accurately reflect environmental stimuli and the central nervous activity including circadian rhythms. Besides, at very low respiratory rates (<7–8 breaths per minute or deep breaths), parasympathetic activity can drive LF power and other indices. Therefore, respiratory rate must be considered, since it is a confounder of short-term measurements. It is also true that the LF/HF ratio has long been considered an index of sympathovagal balance, but this viewpoint has also been strongly criticized because the physiological bases are not clear (Billman, 2013; Shaffer and Ginsberg, 2017). What is more, the study focused on preoperative HRV indices, which changed so dramatically after surgery that they could not reflect the effects of operation and anesthesia exerted on patients.

As reported by Shaffer and Ginsberg (Shaffer and Ginsberg, 2017), the 24-h HRV recordings achieve greater predictive power than short-term measurements. Therefore, our study design provided 24-h ECG recordings postoperatively. Correspondingly, neither the levels of LF nor HF show a difference in our result. There is a significant correlation between POD and VLF, ULF, SDNNI, and TP power, represented as the gold standard for a more accurate evaluation of total circadian HRV (Singh et al., 2018a,b).

The study’s strengths include its prospective nature, blinding of assessors, and use of validated and reliable measures to assess delirium status and HRV parameters.

There are limitations to this work that should be noted. Primarily, the age span of participants included in the cohort is too large. According to the China country assessment report on aging and health, people over 60 are classified as the elderly (No-authors, 2016). Apparently, the young-old patients may not be representative of the entire elderly population. While life expectancy is rising, the health span has not kept up with it (Partridge et al., 2018). The aging process has been linked to the occurrence of chronic diseases and functional impairments, including frailty and metabolic and neurodegenerative diseases (Niccoli and Partridge, 2012; Rivero-Segura et al., 2020). Except for old-old population with hip fractures, a considerable part of the patients who underwent orthopedic surgery were young-old patients who suffered from arthritis. As a common chronic disease in the elderly, an European cross-sectional survey indicates that nearly 50% of elderly patients with symptomatic knee osteoarthritis show fragility (Salaffi et al., 2020). Particularly, fragility is not only a reflection of biologic rather than chronologic age but also an important predisposing factor of delirium (Bersani et al., 2020; Rivero-Segura et al., 2020; Susano et al., 2020).

Specially, due to disadvantageous education (Zhang et al., 2008), cognitive decline in elderly Chinese is more serious. As our research shows, only 25% of participants received high school education or above. Therefore, we set 60 years old as the lower age limit to prevent people who have a young calendar age but experiencing aging from being missed.

In addition, there is a wide spectrum of operative types in this cohort. The study included patients who suffered from hip fracture who were more likely to be older and have with more diseases. A well-known complication of hip fracture surgery is POD, with an incidence up to 56% (Oh et al., 2015). Therefore, we excluded patients with preoperative MMSE score < 17 and preoperative delirium who were more susceptible to POD. Besides, the corresponding baseline HRV of patients should be collected before surgery. Considering the influence of movement and anxiety on HRV before operation (Goessl et al., 2017; Hunt and Saengsuwan, 2018), we only record postoperative long-term HRV, which could reflect the state of patients after surgery more accurately. A more convincing conclusion may require further study to compare predictive power for the occurrences of POD upon HRV of patients before operation and post operation.

What is more, the development of delirium involves the complex inter-relationship between a vulnerable patient with multiple predisposing factors and exposure to noxious insults or precipitating factors (Inouye et al., 2014). Our assessment of the above factors is not comprehensive enough. Finally, due to the small sample size, we cannot adjust for other drugs used in the statistical analyses. Consequently, we excluded the patients who were transferred to the ICU for further treatment because of complex drug use and surroundings. In addition, although generally thought that there is no difference in the incidence of POD between general and regional anesthesia (Mason et al., 2010), we only included patients under spinal anesthesia to eliminate effects on HRV exerted by other anesthetics.

In this prospective cohort study, we investigated the association between postoperative long-term HRV parameters and POD in an orthopedic surgery elderly population. The results showed a negative correlation between VLF, ULF, SDNNI, and TP and incidence and duration of POD. This finding suggests that the occurrence of POD may affected by ANS change and circadian rhythm disorder reflected by postoperative long-term HRV parameters. Thus, future research on how ANS activity and circadian rhythm function affect POD in elderly patients would provide an opportunity to explain relevant pathophysiological factors in POD.

## Data Availability Statement

The original contributions presented in the study are included in the article/Supplementary Material, further inquiries can be directed to the corresponding author.

## Ethics Statement

The studies involving human participants were reviewed and approved by the Medical Ethics Committee of Zhujiang Hospital of Southern Medical University, China. The patients/participants provided their written informed consent to participate in this study.

## Author Contributions

JS and QZ were responsible for the design and implementation of this study, data collection, data statistics, and article writing. BL and ZH are in charge of the patient’s follow-up and neuropsychological testing. DQ analyzes the data of ECG. MH and YP participated in data entry and analysis. QL and PX are responsible for patient data collection. SX designed the project, revised the manuscript, and paid for the project. All authors read and approved the final manuscript.

## Conflict of Interest

The authors declare that the research was conducted in the absence of any commercial or financial relationships that could be construed as a potential conflict of interest.

## References

[B1] Almeida-Santos M. A. Barreto-Filho J. A. Oliveira J. L. Reis F. P. da Cunha Oliveira C. C. Sousa A. C. (2016). Aging, heart rate variability and patterns of autonomic regulation of the heart. *Arch. Gerontol. Geriatr.* 63 1–8. 10.1016/j.archger.2015.11.011 26791165

[B2] Amar D. Fleisher M. Pantuck C. B. Shamoon H. Zhang H. Roistacher N. (1998). Persistent alterations of the autonomic nervous system after noncardiac surgery. *Anesthesiology* 89 30–42. 10.1097/00000542-199807000-00008 9667291

[B3] Battle D. E. (2013). Diagnostic and statistical manual of mental disorders (DSM). *Codas* 25 191–192. 10.1590/s2317-17822013000200017 24413388

[B4] Berntson G. G. Bigger J. T. Jr. Eckberg D. L. Grossman P. Kaufmann P. G. Malik M. (1997). Heart rate variability: origins, methods, and interpretive caveats. *Psychophysiology* 34 623–648. 10.1111/j.1469-8986.1997.tb02140.x 9401419

[B5] Bersani F. S. Canevelli M. Cesari M. Maggioni E. Pasquini M. Wolkowitz O. M. (2020). Frailty Index as a clinical measure of biological age in psychiatry. *J. Affect. Disord.* 268 183–187. 10.1016/j.jad.2020.03.015 32174476

[B6] Billman G. E. (2013). The LF/HF ratio does not accurately measure cardiac sympatho-vagal balance. *Front. Physiol.* 4:26. 10.3389/fphys.2013.00026 23431279 PMC3576706

[B7] Botsva N. Naishtetik I. Khimion L. Chernetchenko D. (2017). Predictors of aging based on the analysis of heart rate variability. *Pacing Clin. Electrophysiol.* 40 1269–1278. 10.1111/pace.13180 28983984

[B8] Carney R. M. Freedland K. E. Stein P. K. Miller G. E. Steinmeyer B. Rich M. W. (2007). Heart rate variability and markers of inflammation and coagulation in depressed patients with coronary heart disease. *J. Psychosom. Res.* 62 463–467. 10.1016/j.jpsychores.2006.12.004 17383498 PMC1924882

[B9] Cerejeira J. Nogueira V. Luis P. Vaz-Serra A. Mukaetova-Ladinska E. B. (2012). The cholinergic system and inflammation: common pathways in delirium pathophysiology. *J. Am. Geriatr. Soc.* 60 669–675. 10.1111/j.1532-5415.2011.03883.x 22316182

[B10] Chadda K. R. Ajijola O. A. Vaseghi M. Shivkumar K. Huang C. L. Jeevaratnam K. (2018). Ageing, the autonomic nervous system and arrhythmia: from brain to heart. *Ageing Res. Rev.* 48 40–50. 10.1016/j.arr.2018.09.005 30300712

[B11] Chouchou F. Khoury S. Chauny J. M. Denis R. Lavigne G. J. (2014). Postoperative sleep disruptions: a potential catalyst of acute pain? *Sleep medicine reviews* 18 273–282. 10.1016/j.smrv.2013.07.002 24074687

[B12] Concato J. Peduzzi P. Holford T. R. Feinstein A. R. (1995). Importance of events per independent variable in proportional hazards analysis. I. Background, goals, and general strategy. *J. Clin. Epidemiol.* 48 1495–1501. 10.1016/0895-4356(95)00510-2 8543963

[B13] Corino V. D. Matteucci M. Cravello L. Ferrari E. Ferrari A. A. Mainardi L. T. (2006). Long-term heart rate variability as a predictor of patient age. *Comput. Methods Programs* 82 248–257. 10.1016/j.cmpb.2006.04.005 16730388

[B14] Dalise A. M. Prestano R. Fasano R. Gambardella A. Barbieri M. Rizzo M. R. (2020). Autonomic nervous system and cognitive impairment in older patients: evidence from long-term heart rate variability in real-life setting. *Front. Aging Neurosci.* 12:40. 10.3389/fnagi.2020.00040 32218729 PMC7079686

[B15] Dasgupta M. Dumbrell A. C. (2006). Preoperative risk assessment for delirium after noncardiac surgery: a systematic review. *J. Am. Geriatr. Soc.* 54 1578–1589. 10.1111/j.1532-5415.2006.00893.x 17038078

[B16] Ernst G. Watne L. O. Rostrup M. Neerland B. E. (2020). Delirium in patients with hip fracture is associated with increased heart rate variability. *Aging Clin. Exp. Res.* 32 2311–2318. 10.1007/s40520-019-01447-5 31916197

[B17] Fadayomi A. B. Ibala R. Bilotta F. Westover M. B. Akeju O. (2018). A systematic review and meta-analysis examining the impact of sleep disturbance on postoperative delirium. *Crit. Care Med.* 46:e1204-12. 10.1097/ccm.0000000000003400 30222634 PMC6274586

[B18] Fisher A. C. Groves D. Eleuteri A. Mesum P. Patterson D. Taggart P. (2014). Heart rate variability at limiting stationarity: evidence of neuro-cardiac control mechanisms operating at ultra-low frequencies. *Physiol. Meas.* 35 309–322. 10.1088/0967-3334/35/2/309 24451405

[B19] Geovanini G. R. Vasques E. R. de Oliveira Alvim R. Mill J. G. Andreão R. V. Vasques B. K. (2020). Age and sex differences in heart rate variability and vagal specific patterns - baependi heart study. *Glob. Heart* 15:71. 10.5334/gh.873 33150136 PMC7583712

[B20] Goessl V. C. Curtiss J. E. Hofmann S. G. (2017). The effect of heart rate variability biofeedback training on stress and anxiety: a meta-analysis. *Psychol. Med.* 47 2578–2586. 10.1017/s0033291717001003 28478782

[B21] Hadase M. Azuma A. Zen K. Asada S. Kawasaki T. Kamitani T. (2004). Very low frequency power of heart rate variability is a powerful predictor of clinical prognosis in patients with congestive heart failure. *Circ. J.* 68 343–347. 10.1253/circj.68.343 15056832

[B22] Huang M. C. Lee C. H. Lai Y. C. Kao Y. F. Lin H. Y. Chen C. H. (2009). Chinese version of the delirium rating scale-revised-98: reliability and validity. *Compr. Psychiatry* 50 81–85. 10.1016/j.comppsych.2008.05.011 19059519

[B23] Hunt K. J. Saengsuwan J. (2018). Changes in heart rate variability with respect to exercise intensity and time during treadmill running. *Biomed. Eng. Online* 17:128. 10.1186/s12938-018-0561-x 30249267 PMC6154948

[B24] Huston J. M. Tracey K. J. (2011). The pulse of inflammation: heart rate variability, the cholinergic anti-inflammatory pathway and implications for therapy. *J. Intern. Med.* 269 45–53. 10.1111/j.1365-2796.2010.02321.x 21158977 PMC4527046

[B25] Inouye S. K. Westendorp R. G. Saczynski J. S. (2014). Delirium in elderly people. *Lancet* 383 911–922. 10.1016/s0140-6736(13)60688-60681 23992774 PMC4120864

[B26] Ji M. Wang J. Yang X. Huang Y. Xiao Y. Wu Y. (2020). Validation of the 3-minute diagnostic interview for CAM-defined Delirium in Chinese older adults. *Geriatr. Nurs.* 42 21–26. 10.1016/j.gerinurse.2020.10.021 33197703

[B27] Kärkelä J. Vakkuri O. Kaukinen S. Huang W. Q. Pasanen M. (2002). The influence of anaesthesia and surgery on the circadian rhythm of melatonin. *Acta Anaesthesiol. Scand.* 46 30–36. 10.1034/j.1399-6576.2002.460106.x 11903069

[B28] Kim M. S. Yoon J. H. Hong J. M. (2018). Early differentiation of dementia with Lewy bodies and Alzheimer’s disease: heart rate variability at mild cognitive impairment stage. *Clin. Neurophysiol.* 129 1570–1578. 10.1016/j.clinph.2018.05.004 29883835

[B29] Kumral D. Schaare H. L. Beyer F. Reinelt J. Uhlig M. Liem F. (2019). The age-dependent relationship between resting heart rate variability and functional brain connectivity. *NeuroImage* 185 521–533. 10.1016/j.neuroimage.2018.10.027 30312808

[B30] Kurtz S. Ong K. Lau E. Mowat F. Halpern M. (2007). Projections of primary and revision hip and knee arthroplasty in the United States from 2005 to 2030. *J. Bone Joint Surgery Am.* 89 780–785. 10.2106/jbjs.f.00222 17403800

[B31] Laborde S. Mosley E. Thayer J. F. (2017). Heart Rate variability and cardiac vagal tone in psychophysiological research - recommendations for experiment planning, data analysis, and data reporting. *Front. Psychol.* 8:213. 10.3389/fpsyg.2017.00213 28265249 PMC5316555

[B32] Lakatta E. G. Levy D. (2003). Arterial and cardiac aging: major shareholders in cardiovascular disease enterprises: Part II: the aging heart in health: links to heart disease. *Circulation* 107 346–354. 10.1161/01.cir.0000048893.62841.f7 12538439

[B33] Lampert R. Bremner J. D. Su S. Miller A. Lee F. Cheema F. (2008). Decreased heart rate variability is associated with higher levels of inflammation in middle-aged men. *Am. Heart. J.* 156 759.e1–759.e7. 10.1016/j.ahj.2008.07.009 18926158 PMC2587932

[B34] Liptzin B. Levkoff S. E. (1992). An empirical study of delirium subtypes. *Br. J. Psychiatry* 161 843–845. 10.1192/bjp.161.6.843 1483173

[B35] Maclullich A. M. Ferguson K. J. Miller T. de Rooij S. E. Cunningham C. (2008). Unravelling the pathophysiology of delirium: a focus on the role of aberrant stress responses. *J. Psychosom. Res.* 65 229–238. 10.1016/j.jpsychores.2008.05.019 18707945 PMC4311661

[B36] Maldonado J. R. (2013). Neuropathogenesis of delirium: review of current etiologic theories and common pathways. *Am. J. Geriatric Psychiatry* 21 1190–1222. 10.1016/j.jagp.2013.09.005 24206937

[B37] Maldonado J. R. (2018). Delirium pathophysiology: an updated hypothesis of the etiology of acute brain failure. *Int. J. Geriatr. Psychiatry* 33 1428–1457. 10.1002/gps.4823 29278283

[B38] Marcantonio E. R. Ngo L. H. O’Connor M. Jones R. N. Crane P. K. Metzger E. D. (2014). 3D-CAM: derivation and validation of a 3-minute diagnostic interview for CAM-defined delirium: a cross-sectional diagnostic test study. *Ann. Intern. Med.* 161 554–561. 10.7326/m14-0865 25329203 PMC4319978

[B39] Marsch S. C. Skarvan K. Schaefer H. G. Naegeli B. Paganoni R. Castelli I. (1994). Prolonged decrease in heart rate variability after elective hip arthroplasty. *Br. J. Anaesth.* 72 643–649. 10.1093/bja/72.6.643 8024911

[B40] Mason S. E. Noel-Storr A. Ritchie C. W. (2010). The impact of general and regional anesthesia on the incidence of post-operative cognitive dysfunction and post-operative delirium: a systematic review with meta-analysis. *J. Alzheimers Dis.* 22(Suppl. 3), 67–79. 10.3233/jad-2010-101086 20858956

[B41] Meagher D. J. Trzepacz P. T. (2000). Motoric subtypes of delirium. *Semin. Clin. Neuropsychiatry* 5 75–85. 10.153/scnp00500075 10837096

[B42] Mulcahy J. S. Larsson D. E. O. Garfinkel S. N. Critchley H. D. (2019). Heart rate variability as a biomarker in health and affective disorders: a perspective on neuroimaging studies. *NeuroImage* 202:116072. 10.1016/j.neuroimage.2019.116072 31386920

[B43] National Bureau of Statistics of China. (2020). *China Statistical Yearbook [M].* China Statistics Press. Available online at: http://www.stats.gov.cn/tjsj/ndsj/2020/indexch.htm

[B44] Neerland B. E. Wyller T. B. Wyller V. B. B. (2019). Autonomic cardiovascular control in older patients with acute infection and delirium: a pilot study of orthostatic stress responses. *BMC Geriatr.* 19:23. 10.1186/s12877-019-1035-0 30683068 PMC6347784

[B45] Niccoli T. Partridge L. (2012). Ageing as a risk factor for disease. *Curr. Biol.* 22 R741–R752. 10.1016/j.cub.2012.07.024 22975005

[B46] No authors listed. (1996). Heart rate variability. Standards of measurement, physiological interpretation, and clinical use. Task Force of the European Society of Cardiology and the North American Society of Pacing and Electrophysiology. *Eur. Heart J.* 17 354–381. 8737210

[B47] No-authors (2016). *China Country Assessment Report on Ageing and Health: World Health Organization^∗^.* Available online at: https://www.who.int/ageing/publications/china-country-assessment/zh/

[B48] Oh E. S. Li M. Fafowora T. M. Inouye S. K. Chen C. H. Rosman L. M. (2015). Preoperative risk factors for postoperative delirium following hip fracture repair: a systematic review. *Int. J. Geriatr. Psychiatry* 30 900–910. 10.1002/gps.4233 25503071 PMC4465414

[B49] Oh E. S. Sieber F. E. Leoutsakos J. M. Inouye S. K. Lee H. B. (2016). Sex differences in hip fracture surgery: preoperative risk factors for delirium and postoperative outcomes. *J. Am. Geriatr. Soc.* 64 1616–1621. 10.1111/jgs.14243 27384742 PMC5038922

[B50] Partridge L. Deelen J. Slagboom P. E. (2018). Facing up to the global challenges of ageing. *Nature* 561 45–56. 10.1038/s41586-018-0457-8 30185958

[B51] Perseguini N. M. Verlengia R. Milan J. C. Minatel V. Rehder-Santos P. Takahashi A. C. (2015). Cardiac autonomic modulation, C-reactive protein or telomere length: which of these variables has greater importance to aging? *Int. J. Cardiol.* 178 79–81. 10.1016/j.ijcard.2014.10.123 25464224

[B52] Riedel B. Browne K. Silbert B. (2014). Cerebral protection: inflammation, endothelial dysfunction, and postoperative cognitive dysfunction. *Curr. Opin. Anaesthesiol.* 27 89–97. 10.1097/aco.0000000000000032 24300462

[B53] Riganello F. Larroque S. K. Di Perri C. Prada V. Sannita W. G. Laureys S. (2019a). Measures of CNS-autonomic interaction and responsiveness in disorder of consciousness. *Front. Neurosci.* 13:530. 10.3389/fnins.2019.00530 31293365 PMC6598458

[B54] Riganello F. Prada V. Soddu A. di Perri C. Sannita W. G. (2019b). Circadian rhythms and measures of CNS/autonomic interaction. *Int. J. Environ. Res. Public Health* 16:2336. 10.3390/ijerph16132336 31269700 PMC6651187

[B55] Rivero-Segura N. A. Bello-Chavolla O. Y. Barrera-Vázquez O. S. Gutierrez-Robledo L. M. Gomez-Verjan J. C. (2020). Promising biomarkers of human aging: in search of a multi-omics panel to understand the aging process from a multidimensional perspective. *Ageing Res. Rev.* 64:101164. 10.1016/j.arr.2020.101164 32977058

[B56] Salaffi F. Di Carlo M. Carotti M. Farah S. Giovagnoni A. (2020). Frailty prevalence according to the Survey of Health, Ageing and Retirement in Europe-Frailty Instrument (SHARE-FI) definition, and its variables associated, in patients with symptomatic knee osteoarthritis: findings from a cross-sectional study. *Aging Clin. Exp. Res* 10.1007/s40520-020-01667-0 Online ahead of print 32734577 PMC8203526

[B57] Schmidt H. Müller-Werdan U. Hoffmann T. Francis D. P. Piepoli M. F. Rauchhaus M. (2005). Autonomic dysfunction predicts mortality in patients with multiple organ dysfunction syndrome of different age groups. *Crit. Care Med.* 33 1994–2002. 10.1097/01.ccm.0000178181.91250.99 16148471

[B58] Scott B. K. (2015). Disruption of circadian rhythms and sleep in critical illness and its impact on the development of delirium. *Curr. Pharm. Des.* 21 3443–3452. 10.2174/1381612821666150706110656 26144937

[B59] Scott J. E. Mathias J. L. Kneebone A. C. (2015). Incidence of delirium following total joint replacement in older adults: a meta-analysis. *Gen. Hosp. Psychiatry* 37 223–229. 10.1016/j.genhosppsych.2015.02.004 25774049

[B60] Shaffer F. Ginsberg J. P. (2017). An overview of heart rate variability metrics and norms. *Front. Public Health* 5:258. 10.3389/fpubh.2017.00258 29034226 PMC5624990

[B61] Shaffer F. McCraty R. Zerr C. L. (2014). A healthy heart is not a metronome: an integrative review of the heart’s anatomy and heart rate variability. *Front. Psychol.* 5:1040. 10.3389/fpsyg.2014.01040 25324790 PMC4179748

[B62] Singh N. Moneghetti K. J. Christle J. W. Hadley D. Froelicher V. Plews D. (2018a). Heart rate variability: an old metric with new meaning in the era of using mhealth technologies for health and exercise training guidance. Part two: prognosis and training. *Arrhythm. Electrophysiol. Rev.* 7 247–255. 10.15420/aer.2018.30.2 30588312 PMC6304793

[B63] Singh N. Moneghetti K. J. Christle J. W. Hadley D. Plews D. Froelicher V. (2018b). Heart rate variability: an old metric with new meaning in the era of using mhealth technologies for health and exercise training guidance. Part one: physiology and methods. *Arrhythm. Electrophysiol. Rev.* 7 193–198. 10.15420/aer.2018.27.2 30416733 PMC6141929

[B64] Subramaniyan S. Terrando N. (2019). Neuroinflammation and perioperative neurocognitive disorders. *Anesth. Analg.* 128 781–788. 10.1213/ane.0000000000004053 30883423 PMC6437083

[B65] Susano M. J. Grasfield R. H. Friese M. Rosner B. Crosby G. Bader A. M. (2020). Brief preoperative screening for frailty and cognitive impairment predicts delirium after spine surgery. *Anesthesiology* 133 1184–1191. 10.1097/aln.0000000000003523 32898243 PMC7657972

[B66] Tan J. P. H. Beilharz J. E. Vollmer-Conna U. Cvejic E. (2019). Heart rate variability as a marker of healthy ageing. *Int. J. Cardiol.* 275 101–103. 10.1016/j.ijcard.2018.08.005 30104034

[B67] Taylor J. A. Carr D. L. Myers C. W. Eckberg D. L. (1998). Mechanisms underlying very-low-frequency RR-interval oscillations in humans. *Circulation* 98 547–555. 10.1161/01.cir.98.6.547 9714112

[B68] Thayer J. F. Hansen A. L. Saus-Rose E. Johnsen B. H. (2009). Heart rate variability, prefrontal neural function, and cognitive performance: the neurovisceral integration perspective on self-regulation, adaptation, and health. *Ann. Behav. Med.* 37 141–153. 10.1007/s12160-009-9101-z 19424767

[B69] Trzepacz P. T. Franco J. G. Meagher D. J. Lee Y. Kim J. L. Kishi Y. (2018). Delirium phenotype by age and sex in a pooled data set of adult patients. *J. Neuropsychiatry Clin. Neurosci.* 30 294–301. 10.1176/appi.neuropsych.18020024 30045679

[B70] Tsuji H. Venditti F. J. Jr. Manders E. S. Evans J. C. Larson M. G. Feldman C. L. (1994). Reduced heart rate variability and mortality risk in an elderly cohort. The framingham heart study. *Circulation* 90 878–883. 10.1161/01.cir.90.2.878 8044959

[B71] Ulmer C. S. Hall M. H. Dennis P. A. Beckham J. C. Germain A. (2018). Posttraumatic stress disorder diagnosis is associated with reduced parasympathetic activity during sleep in US veterans and military service members of the Iraq and Afghanistan wars. *Sleep* 41:zsy174. 10.1093/sleep/zsy174 30169878 PMC6289238

[B72] van Meenen L. C. van Meenen D. M. de Rooij S. E. ter Riet G. (2014). Risk prediction models for postoperative delirium: a systematic review and meta-analysis. *J. Am. Geriatr. Soc.* 62 2383–2390. 10.1111/jgs.13138 25516034

[B73] Vitale J. A. Bonato M. La Torre A. Banfi G. (2019). Heart rate variability in sport performance: do time of day and chronotype play a role? *J. Clin. Med.* 8:723. 10.3390/jcm8050723 31117327 PMC6571903

[B74] Weinstein S. M. Poultsides L. Baaklini L. R. Morwald E. E. Cozowicz C. Saleh J. N. (2018). Postoperative delirium in total knee and hip arthroplasty patients: a study of perioperative modifiable risk factors. *Br. J. Anaesth.* 120 999–1008. 10.1016/j.bja.2017.12.046 29661417

[B75] Williams D. P. Koenig J. Carnevali L. Sgoifo A. Jarczok M. N. Sternberg E. M. (2019). Heart rate variability and inflammation: a meta-analysis of human studies. *Brain Behav. Immun.* 80 219–226. 10.1016/j.bbi.2019.03.009 30872091

[B76] Zhang C. Feng J. Wang S. Gao P. Xu L. Zhu J. (2020). Incidence of and trends in hip fracture among adults in urban China: a nationwide retrospective cohort study. *PLoS Med.* 17:e1003180. 10.1371/journal.pmed.1003180 32760065 PMC7410202

[B77] Zhang Z. Gu D. Hayward M. D. (2008). Early life influences on cognitive impairment among oldest old Chinese. *J Gerontol. B Psychol. Sci. Soc. Sci.* 63 S25–S33. 10.1093/geronb/63.1.s25 18332198

