# Supplementary material for: Association Between Postoperative Long-Term Heart Rate Variability and Postoperative Delirium in Elderly Patients Undergoing Orthopedic Surgery: A Prospective Cohort Study
Source: Front Aging Neurosci. 2021 May 31;13:646253. doi: 10.3389/fnagi.2021.646253 (PMC8200544; doi:10.3389/fnagi.2021.646253)
Supplement: Supplementary file 1 [file Data_Sheet_1.docx]

Supplementary Material

# Supplementary Tables

## Supplementary Table 1

## SUPPLEMENTARY TABLE 1 | Descriptive characteristics of the patients with POD, stratified by the subtype of delirium.

|  | Hyperactive subtype, *n* = 18, (30.0%) | Hypoactive subtype, *n* = 17, (28.33%) | Mixed subtype, *n* = 25, (41.67%) | *P* |
| --- | --- | --- | --- | --- |
| Age (years) | 75.44 ± 7.01 | 72.59 ± 10.27 | 73.68 ± 7.32 | 0.580 |
| Sex |  |  |  | 0.362 |
| female | 10 (55.60%) | 13 (76.50%) | 18 (72.00%) |  |
| male | 8 (44.40%) | 4 (23.50%) | 7 (28.00%) |  |
| Education level |  |  |  | 0.710 |
| < high school | 13 (72.20%) | 14 (82.40%) | 18 (72.00%) |  |
| >= high school | 5 (27.80%) | 3 (17.60%) | 7 (28.00%) |  |
| BMI , kg/m^2^ | 23.45 ± 3.66 | 24.44 ± 3.63 | 24.01 ± 4.27 | 0.752 |
| Hb (g/L) | 113.39 ± 18.73 | 118.88 ± 16.57 | 115.00 ± 18.55 | 0.673 |
| ASA PS |  |  |  | 0.479 |
| 1 or 2 | 11 (61.10%) | 12 (70.60%) | 13 (52.00%) |  |
| 3 | 7 (38.90%) | 5 (29.40%) | 12 (48.00%) |  |
| CCI | 1.28 ± 1.49 | 1.29 ± 1.45 | 1.08 ± 1.15 | 0.843 |
| Type of surgery |  |  |  | 0.113 |
| THA or RTHA | 8 (44.40%) | 2 (11.80%) | 10 (40.00%) |  |
| TKA or RTKA | 6 (33.30%) | 10 (58.80%) | 6 (24.00%) |  |
| Hip fracture repair or FSF surgery | 4 (22.20%) | 5 (29.40%) | 9 (36.00%) |  |
| Duration of surgery (min) | 157.78 ± 62.48 | 145.00 ± 41.12 | 108.20 ± 31.98 | 0.002 |
| Midazolam (mg) | 1.50 ± 0.71 | 1.41 ± 0.59 | 1.72 ± 0.78 | 0.350 |
| Equivalent morphine (mg) | 72.63 ± 49.28 | 39.95 ± 39.25 | 66.80 ± 44.16 | 0.074 |

*Data are reported as mean ± SD or n (percentage). BMI, body mass index; Hb, hemoglobin; ASA PS, physical status according to the American Society of Anesthesiologists; CCI, Charlson Comorbidity Index; THA, total hip arthroplasty; RTHA, revision total hip anhroplasty; TKA, total kneearthroplasty; RTKA, revision total knee arthroplasty; FSF surgery, Femoral Shaft Fracture surgery*

## Supplementary Table 2

**SUPPLEMENTARY TABLE 2 |** HRV parameters of the patients with POD, stratified by the subtype of delirium.

|  | Hyperactive subtype, *n* = 18, (30.0%) | Hypoactive subtype, *n* = 17, (28.33%) | Mixed subtype, *n* = 25, (41.67%) | *P* |
| --- | --- | --- | --- | --- |
| HR mean (bmp) | 77.89 ± 14.88 | 74.41 ± 12.18 | 74.76 ± 12.12 | 0.671 |
| SDNN (ms) | 63.50 (48.25, 83.25) | 88.00 (63.50, 109.00) | 77.00 (59.00, 112.00) | 0.112 |
| SDNNI (ms) | 25.50 (15.25, 31.25) | 33.00 (21.50, 38.00) | 29.00 (20.00, 39.00) | 0.156 |
| RMSSD (ms) | 17.50 (9.00, 23.25) | 20.00 (14.00, 32.50) | 22.00 (13.50, 29.00) | 0.200 |
| HF (nu) | 35.76 (31.04, 49.75) | 35.41 (26.99, 48.99) | 35.59 (30.61, 47.05) | 0.906 |
| LF (nu) | 51.44 (42.80, 59.11) | 60.00 (46.72, 67.77) | 53.94 (42.66, 59.46) | 0.301 |
| VLF (ms) | 250.85 (127.15, 487.93) | 414.60 (179.30, 562.55) | 368.80 (172.35, 597.30) | 0.361 |
| ULF (ms) | 10.60 (5.50, 22.68) | 16.30 (7.90, 24.80) | 15.20 (6.75, 26.70) | 0.567 |
| TP (ms) | 416.20 (172.10, 714.78) | 819.70 (324.55, 1165.40) | 666.80 (272.10, 1167.40) | 0.105 |

*Data are reported as mean ± SD or median (interquartile range).HR, heart rate; bpm, beats per minutes; SDNN, standard deviation of all normal to normal intervals; SDNNI, mean of the standard deviations of all the NN intervals for each 5-min segment of a 24-h HRV recording; RMSSD, square root of the mean of the sum of the squares of differences between adjacent normal to normal intervals; HF, high frequency; LF, low frequency; VLF, very low frequency; ULF, ultra low frequency; TP, total power.*

- 1. **Supplementary Table 3**

**SUPPLEMENTARY TABLE 3 |** Spearman’s correlation coefficients between HRV parameters and POD, stratified by the age.

|  | Young-old, *n* = 251, (85.37%) | | Old-old, *n* = 43, (14.63%) | |
| --- | --- | --- | --- | --- |
| Variables | ρ | *P* | ρ | *P* |
| SDNN (ms) | -0.093 | 0.142 | 0.184 | 0.237 |
| SDNNI (ms) | -0.162 | 0.010 | 0.074 | 0.638 |
| RMSSD (ms) | -0.079 | 0.213 | -0.016 | 0.921 |
| HF (nu) | 0.014 | 0.827 | -0.205 | 0.186 |
| LF (nu) | -0.029 | 0.652 | 0.120 | 0.443 |
| VLF (ms) | -0.178 | 0.005 | 0.062 | 0.693 |
| ULF (ms) | -0.192 | 0.002 | 0.143 | 0.359 |
| TP (ms) | -0.156 | 0.014 | 0.151 | 0.333 |

*SDNN, standard deviation of all normal to normal intervals; SDNNI, mean of the standard deviations of all the NN intervals for each 5-min segment of a 24-h HRV recording; RMSSD, square root of the mean of the sum of the squares of differences between adjacent normal to normal intervals; HF, high frequency; LF, low frequency; ULF, ultra-low frequency; TP, total power.*

- 1. **Supplementary Table 4**

**SUPPLEMENTARY TABLE 4 |** HRV parameters of the cohort, stratified by age.

|  | Young, *n* = 251, (85.37%) | Old, *n* = 43, (14.63%) | *P* |
| --- | --- | --- | --- |
| SDNN (ms) | 80.00 (61.50, 101.00) | 70 (54.50, 95.50) | 0.101 |
| RMSSD (ms) | 19.00 (14.00, 26.50) | 22.00 (16.00, 33.00) | 0.109 |
| HF (nu) | 34.66 (27.48, 43.33) | 47.05 (38.14, 54.91) | ＜0.001 |
| LF (nu) | 57.20 (48.30, 62.43) | 47.77 (34.81, 53.72) | ＜0.001 |
| VLF (ms) | 485.50 (280.45, 694.95) | 278.00 (157.75, 491.15) | ＜0.001 |
| ULF (ms) | 21.70 (12.65, 31.25) | 11.50 (6.40, 21.10) | ＜0.001 |
| TP (ms) | 786.50 (468.55, 1201.75) | 496.70 (251.20, 1001.90) | 0.026 |
